# Influenza vaccine recommendations and coverage (2018–2023): a foundation for pandemic preparedness and response

**DOI:** 10.1016/j.vaccine.2026.128391

**Published:** 2026-04-11

**Authors:** Margaux M.I. Meslé, Pernille Jorgensen, Amal Barakat, Belinda L. Herring, Francisco Nogareda, Marcela Contreras, Martha Velandia-Gonzalez, Pushpa Wijesinghe, Reena Doshi, Ioana Ghiga, Randie Gibson, Laure Dumolard, Donald J Brooks, Oluwatosin W. Akande, Philipp Lambach, Vanessa Cozza, Ann C. Moen, Stefano Tempia, Shoshanna Goldin

**Affiliations:** a World Health Organization, Regional Office for Europe, Denmark; b World Health Organization, Regional Office for Eastern Mediterranean, Egypt; c World Health Organization, Regional Office for Western Pacific, Philippines; d Pan American Health Organization, Comprehensive Immunization Program (CIM), United States of America; e World Health Organization, Regional Office for South-East Asia, India; f World Health Organization, Regional Office for Africa, Republic of the Congo; g World Health Organization, Headquarters, Epidemic and Pandemic Management, Switzerland; h World Health Organization, Headquarters, Immunization, Vaccines, and Biologicals, Switzerland; i The Task Force for Global Health, United States of America

**Keywords:** Influenza vaccination, Vaccine recommendations, Vaccination coverage, Vaccine doses

## Abstract

Seasonal influenza causes millions of severe illnesses and deaths globally, especially in vulnerable populations. Influenza vaccines reduce morbidity and mortality, if given to vulnerable populations prior to the peak influenza season. WHO regularly analyzes data on seasonal influenza vaccination provided by Member States and Territories (MST) to monitor the global status of vaccination policies, programmes, and coverage as national seasonal influenza vaccination programmes have been repeatedly documented to provide a platform for pandemic preparedness and response. This manuscript examines global influenza vaccination coverage between 2018 and 2023.

We analyzed the recommendations and vaccination coverage data as reported by MST to the WHO-UNICEF Joint Reporting Form on Immunization (JRF) together with income classifications from the World Bank to report on recommendations and vaccination coverage per WHO region and income groups over time. We undertook two linear regressions to understand associations between higher vaccination coverage rates in older adults and independent variables.

Globally, MST reporting to JRF increased over time (from 84% to 93%). While reporting of influenza vaccination recommendations by target group has remained steady (from 65% to 64%), reporting of coverage data increased slightly (from 51% to 53%), with variations between WHO regions. Among MST who completed the JRF, recommendations for vaccinating Health and Care Workers (HCWs) (63%) and older adults (62%) remain the most frequent, with median coverage of HCWs higher than that for older adults in four WHO regions. Median coverage for pregnant women increased substantially in WPR. Gross domestic product was the only statistically significant predictor for high coverage in older adults.

Since 2018, a growing number of MST have issued national recommendations for seasonal vaccination and improved coverage of seasonal influenza vaccination, although significant differences remain between WHO regions. Continuing this positive trajectory will keep strengthening national preparedness for and resilience to future respiratory pathogen pandemics.

## Highlights

• More MST reported seasonal influenza vaccination data annually to WHO.
• More MST introduced seasonal influenza vaccination policies and reached higher coverage.
• The COVID-19 pandemic impacted the number of doses distributed.
• National Gross Domestic Product predicted higher vaccination coverage in older adults.
• Vaccination for pregnant women has increased significantly between 2019 and 2023.

## 1 Introduction

Seasonal influenza vaccination reduces morbidity and mortality during annual epidemics and supports global pandemic preparedness [1]. The World Health Organization (WHO) recommends annual influenza vaccination for older adults, people with underlying conditions and comorbidities, pregnant women, and Health and Care Workers (HCWs)) [2]. At the 56th World Health Assembly (2003), Member States unanimously agreed to prioritize seasonal influenza vaccination and reach a coverage target of 75% for older adults and persons with chronic conditions [3]. Within the Global Influenza Strategy 2019–2030 and the Pandemic Influenza Preparedness Framework's High Level Implementation Plan III (2024–2030), seasonal influenza vaccination is recognized as a global priority [4]. Recognizing the importance of seasonal influenza vaccination for all countries and the role that seasonal influenza vaccination plays as a primary example of life course immunization, the Immunization Agenda 2030 (IA2030) monitors influenza vaccine introduction in low and middle-income countries [2], [5]. WHO recommends all countries consider implementing an influenza vaccination programme [2].

Seasonal influenza vaccines have been used for more than eighty years to reduce illness, hospitalizations, and deaths globally. While countries across income classifications conduct seasonal influenza vaccination, national programmes are more prevalent in high-income and upper-middle-income countries.

Member States and territories (henceforth referred to as MST) annually provide data through the WHO-UNICEF Joint Reporting Form on Immunization (JRF). WHO regularly analyzes the global status of vaccination policies, programmes, and coverage [6], [7], [8], [9]. In addition, this analysis provides an overview of global seasonal influenza vaccination coverage reporting trends to inform the development of the upcoming WHO manual on monitoring and reporting of seasonal influenza, COVID-19, and Respiratory Syncytial Virus (RSV) [10].

## 2 Methods

Expanded Programme on Immunization (EPI) managers and national focal points for influenza vaccination report about their previous year or previous influenza season data annually to the JRF (World Health Organization, Immunization dashboard, 2025); for example, 2023 calendar year data or 2022–2023 seasonal data were reported in 2024. This analysis collated data from 196 reporting MST, which includes 194 WHO Member States and two reporting entities: the occupied Palestinian territory, including east Jerusalem, and Kosovo (in accordance with United Nations Security Council resolution 1244 (1999)). The analysis examines data from 2018 to 2023 across all six WHO Regions (African (AFR) (*n* = 47 MST), Americas (AMR) (*n* = 35 MST), Eastern Mediterranean (EMR) (*n* = 22 MST), European (EUR) (*n* = 54 MST), South-East Asian (SEAR) (*n* = 11 MST),^1^ and Western Pacific (WPR) (*n* = 27 MST)) (Supplementary table 1). The data were downloaded from the WHO Immunization Data Warehouse on 1 May 2025 [11]. This manuscript analyzes trends in influenza vaccine recommendations, target population size estimates (denominators), vaccination uptake (numerators) and coverage (as a percentage of the target population), and number of doses distributed within each MST.

In 2022, WHO revised the SAGE guidance on seasonal influenza vaccination and updated the recommended target groups to be older adults, pregnant women, HCWs, and persons with chronic conditions [2]. This guidance encourages MST to consider vaccination of other groups based on national priorities, resources, and feasibility. Many countries have national recommendations for seasonal influenza vaccination for children; there are wide variations across countries in the ages recommended for vaccination. In accordance with the 2022 SAGE guidance, we analyzed seasonal influenza vaccine recommendations for the SAGE recommended target groups. We also analyzed vaccination coverage data for older adults, pregnant women, and HCWs, as these groups had more complete data and comparable recommendations.

Additional data sources were also included in the current analysis, including World Bank 2024 country income classification data, downloaded in October 2024 [12], using the latest historical grouping available (2016) for Venezuela and Niue. National population estimates by five-year age band were downloaded from the World Population Prospects of the United Nations (UN) Department of Economic and Social Affairs Population Division database on 2 December 2024 [13]. World Bank Gross Domestic Product data were downloaded on 4 April 2025 [14]. As MST did not always report denominators in the JRF, we used denominators from other sources to include additional MST in the analysis. If the age group was not provided for older adults, it was assumed to be ≥65 years, as this is the age range used by the majority of MST.

Broader consolidated age groupings corresponding to country vaccination recommendations (for example, ≥65 years) were calculated by summing smaller age groups. These estimates were used as denominators for coverage calculations if MST did not provide denominator information for three or more years. If denominators were missing for one or two years, the previously reported denominators were used (applicable to Argentina, Bhutan, Bulgaria, Guatemala, Jamaica, Laos, Mauritius, Saudi Arabia, and Serbia). Calculated denominators for older adults, matching the reported age groups, were derived from UN population estimates for Andorra, Bahamas, Bahrain, Grenada, Luxembourg, Saint Kitts and Nevis, Suriname, Trinidad and Tobago, Tunisia, Turkmenistan, and Uzbekistan for all years. Denominator data for pregnant women and HCWs, where missing, were collected from the UN population estimates for live births and International Labour Organization, respectively, on 20 March 2022 [15], [16]. Vaccination coverage data (either reported or calculated) above 100% were removed from the analysis.

Two linear regression analyses were conducted to determine if there was an association between higher vaccination coverage rates for older adults: a time series as a mixed effects linear regression model (with clustering by country) and a linear regression model looking at 2023 only. These analyses were conducted only for older adults as there was insufficient coverage data available for the other target groups. The independent variables were: 10.13039/100004421World Bank income classification; 10.13039/100004421World Bank Gross Domestic Product (GDP); presence of a functioning National Immunization Technical Advisory Group (NITAG); presence of a formal national influenza vaccination policy; presence of influenza vaccination recommendations; payment schemes for influenza vaccinations; influenza vaccine types used; presence of influenza vaccination in private and/or public sectors; programme responsible for delivering influenza vaccination (2023 only); support from external financial sources (such as the Task Force for 10.13039/100006090Global Health's Partnership for International Vaccines Initiative (PIVI)) and reporting of universal influenza vaccination coverage (meaning, presence of a national recommendation for vaccination of all individuals aged ≥six months). The source of all the independent variables was WHO (JRF, Global Health Observatory) or the World Bank (income classification and GDP). For the univariate analysis, the following predictors had a *p* < 0.2 (and were tested for significance): GDP; presence of a seasonal influenza vaccination policy in the private and/or public sectors; presence of a functioning NITAG; and the programme responsible for delivering influenza vaccination (data for 2023 only). These predictors were assessed for statistical significance in the multivariable regression.

MST were considered as having responded to the JRF form if they responded (even by replying ‘no data’ or ‘not relevant’) to at least one of the following questions: ‘Were influenza vaccines available this season?’, ‘Does the country have a formal national (governmental) influenza vaccination policy?’ and ‘Was seasonal influenza vaccination recommended for at least one target group?’. Reporting was deemed consistent if the MST replied to at least one of these questions for at least four of the six years considered.

Data were aggregated by WHO region, income classification, and at the global level. Analyses were conducted using R statistical software (version 4.3.1) [17], with packages tidyverse, lmer and ggplot2.

## 3 Results

### 3.1 Consistency and quality of reporting

#### 3.1.1 Data completeness

**Fig. 1:**
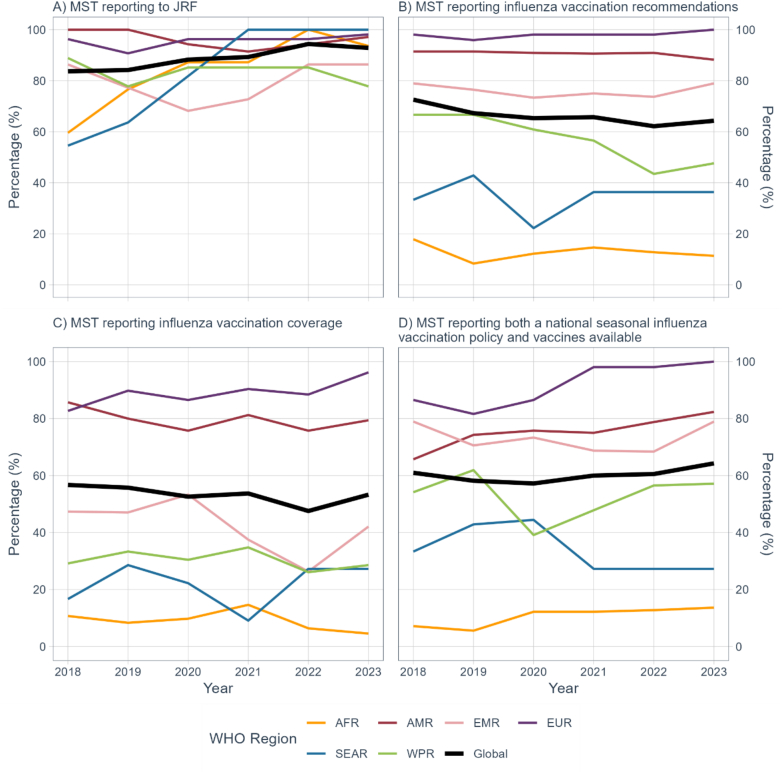
Trends in reporting by MST per year and WHO region, A) of MST completing the influenza vaccination JRF questions; B) among MST who returned a completed influenza JRF, percentage reporting influenza vaccination recommendations for at least one target group; C) among MST who returned a completed influenza JRF, percentage reporting vaccination coverage data for at least one target group; and D) among MST who returned a completed influenza JRF, percentage reporting having an influenza policy and vaccines available.

Between 2018 and 2023, the number of MST reporting on the influenza vaccination indicators identified in the methods section increased. Of the 196 MST included in this analysis, 84% (*n* = 164) completed the 2018 JRF compared to 93% (*n* = 182) in 2023. AFR and SEAR made the largest reporting gains: from 60% (*n* = 28) in 2018 to 94% (*n* = 44) in 2023 and from 55% (*n* = 6) in 2018 to 100% (*n* = 11) in 2023, respectively. However, WPR decreased between 2018 (89%, *n* = 24) and 2023 (78%, *n* = 21). The other three regions maintained stable reporting rates: from 100% (*n* = 35) to 97% (*n* = 34) in AMR; and from 96% (*n* = 52) to 98% (*n* = 53) in EUR; and from 86% (*n* = 19) to 86% (*n* = 19) in EMR (Fig. 1A, Supplementary Fig. 1).

#### 3.1.2 Recommendations for at least one target group

The percentage of MST that reported having seasonal influenza vaccination recommendations remained stable, from 119 MST of 164 MST in 2018 to 117 of 182 MST) in 2023. Regionally, trends in recommendations for at least one target group have varied substantially. For example, MST in AFR reporting a recommendation for seasonal influenza vaccination remained between 14% (*n* = 45) and 11% (*n* = 5) from 2018 to 2023. In WPR, there was a drop in the number of MST that submitted influenza vaccination data to the JRF; as a result, the percentage of MST reporting a recommendation for influenza vaccination decreased from 67% (*n* = 16) in 2018 to 48% (*n* = 10) in 2023. AMR saw a decrease in percentage of MST reporting recommendations for at least one target group, from 91% (*n* = 32) to 88% (*n* = 30). EUR and SEAR both saw an increase in reporting national recommendations from 94% (*n* = 51) to 98% (*n* = 53) and 9% (*n* = 1) and 36% (*n* = 4), respectively, whereas EMR remained constant at 68% (*n* = 15) (Fig. 1B).

#### 3.1.3 Reporting of coverage data

Globally, there was a slight increase in the number of MST reporting coverage data (either numerator or calculated coverage) for at least one target group, 93 MST in 2018 to 97 MST in 2023 (Fig. 1C). EUR and SEAR showed an increasing trend in the percentage of MST reporting coverage data for at least one target group over time, with an increase from 83% (*n* = 43) in 2018 to 96% (*n* = 51) in 2023 in EUR and from 17% (*n* = 1) in 2018 to 27% (*n* = 3) in 2023 in SEAR. In AMR, the percentage of MST reporting coverage data decreased from 86% (*n* = 30) in 2018 to 79% (*n* = 27) in 2023 (Fig. 1C).

#### 3.1.4 Presence of national policy and vaccines available

From 2018 to 2023, the number of MST with both formal national policies for influenza vaccination and influenza vaccines available in the public and/or private sector increased from 61% (*n* = 100) MST to 64% (*n* = 117) MST. Correspondingly, the percentage of MST reporting that they did not have vaccination policies but had vaccines available has decreased from 12% (*n* = 20) MST in 2018 to 8% (*n* = 15) MST in 2023. The number of MST reporting having a policy but no vaccines available remained constant, with three MST in 2018 compared to four MST in 2023 (Fig. 1D).

### 3.2 Trends over time

#### 3.2.1 Recommendations

##### 3.2.1.1 Older adults

**Fig. 2:**
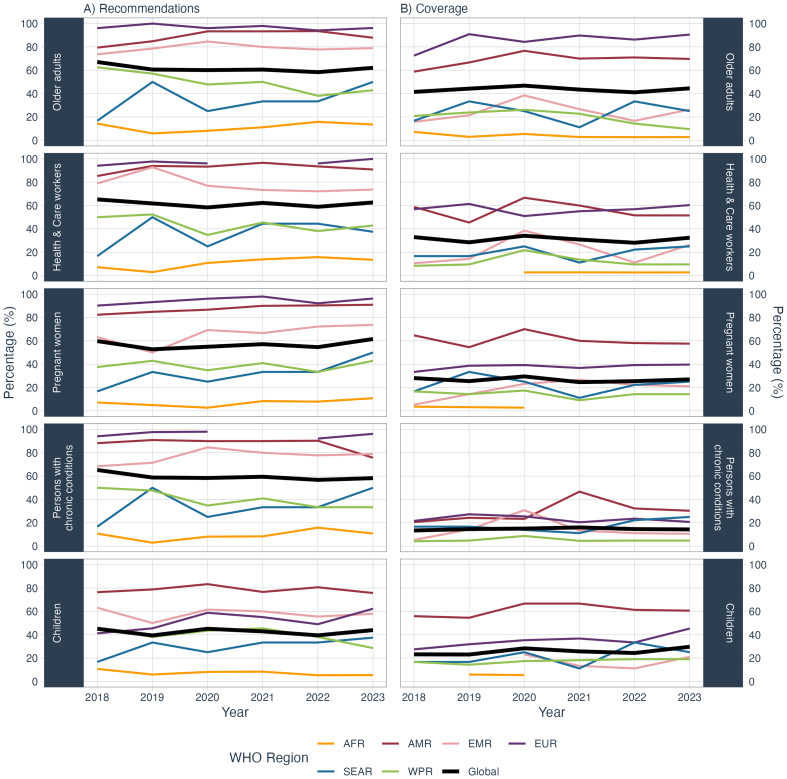
Percentage of MST (A) reporting having recommendations and (B) reporting coverage data for influenza vaccination for WHO-recommended target groups by year and WHO region.

The number of MST reporting recommendations on seasonal influenza vaccination for their older adult population remained steady, with 110 MST in 2018 compared to 113 MST in 2023 (Fig. 2). In four WHO regions (AMR, EMR, EUR, and SEAR), the percentage of MST with recommendations for older adults increased during this period: from 77% to 85% in AMR; from 74% to 79% in EMR; from 94% to 96% in EUR; and from 17% to 36% in SEAR (Fig. 2, Supplementary Table 2).

Of the MST that provided both information on national recommendations for seasonal influenza vaccination for older adults and information on the recommended age group in 2023 (*n* = 85), the majority (66%, *n* = 56) reported recommendations for people ≥65 years. This was an increase from 57% (*n* = 37) MST reporting recommendations for people ≥65 years in 2018. In comparison, 34% (*n* = 22) in 2018 and 28% (*n* = 24) in 2023 MST reported recommending vaccination for their older adults aged ≥60 years, whereas up to 4% (*n* = 4) reported recommending vaccination for people aged ≥50 years.

##### 3.2.1.2 Health and care workers

The number of MST with recommendations for HCWs increased from 107 in 2018 to 114 in 2023. The largest percentage changes were seen in EUR recorded the highest percentage of MST in 2023 (100%, *n* = 53) up from 92% in 2018 (*n* = 48); in WPR, the percentage of MST reporting a recommendation for HCWs decreased from 50% (*n* = 12) in 2018 to 43% (*n* = 9) in 2023. AFR, AMR and SEAR all saw increased percentages of recommendations reporting over time (from 7% to 11%; from 83% to 88%; and 17% to 27%, respectively), whereas EMR experienced a slight decrease (from 79% (*n* = 15) to 74% (*n* = 14)) (Fig. 2, Supplementary Table 2). While most countries with these recommendations provided influenza vaccination for all HCWs (67%, *n* = 39 in 2023), a small number of countries limited this recommendation to HCWs in contact with patients or with patients at risk of complications from influenza infection.

##### 3.2.1.3 Pregnant women

Globally, the number of MST with recommendations for pregnant women increased from 98 in 2018 to 112 in 2023. Regionally, EUR had the highest percentage of MST recommending vaccination of pregnant women in 2023 (96%, *n* = 51). No region reported a decreasing percentage of MST having recommendations for pregnant women (Fig. 2, Supplementary Table 2). One MST reported having recommendations for pregnant women, but vaccines only available in the private sector for three consecutive years.

Of the MST who provided detailed information on the recommendations for pregnant women (18 in 2018 compared to 62 in 2023), there was a substantial increase in the percentage of MST recommending vaccination for all pregnant women, from 11% (*n* = 2) in 2018 to 73% (*n* = 48) in 2023. The criteria for recommending seasonal influenza vaccines to pregnant women varied across MST: two reported recommending seasonal influenza vaccination to those with chronic conditions only; 12 to pregnant women from their second trimester if healthy (of which two recommended vaccination in the first trimester if the mother was at risk of complications); and 48 to all interested in receiving the vaccine.

##### 3.2.1.4 Children

The number of MST reporting recommendations for children increased slightly from 76 in 2018 to 80 in 2023. Regionally, AMR had the highest percentage of MST with recommendations for children in 2023 (74%, *n* = 25) with the percentage of MST reporting staying steady. EUR and SEAR reported an increasing percentage of MST having recommendations for children between 2018 and 2023: from 40% (*n* = 21) to 62% (*n* = 33); and from 17% (*n* = 1) to 27% (*n* = 3), respectively. The other three regions all reported a decrease: from 11% to 5% in AFR; from 63% to 58% in EMR and from 46% to 29% in WPR (Fig. 2, Supplementary Table 2). One MST reported having recommendations for children, but no vaccines were available for three consecutive years.

Of the MST who provided detailed information on recommendations for children (*n* = 25 in 2018 and *n* = 75 in 2023), an increasing percentage reported recommending vaccination for children aged ≥6 months, from 68% (*n* = 17) in 2018 to 78% (*n* = 59) in 2023.^2^ MST reported a range of ages recommended for childhood influenza vaccination, from 6 months to 19 years. In addition, some MST recommend seasonal influenza vaccination only for children with underlying comorbidities and/or chronic illness.

##### 3.2.1.5 Persons with chronic conditions

The number of MST reporting recommendations for persons with chronic conditions remained steady with 107 in 2018 compared to 106 in 2023. Regionally, EUR had the highest percentage of MST with recommendations for persons with chronic conditions (96%, *n* = 51). Four regions reported an increasing percentage of MST having recommendations for persons with chronic conditions between 2018 and 2023 (Fig. 2, Supplementary Table 2).

In 2018, one MST recommended vaccination for children with chronic conditions only, nine reported recommending for adults only, and 97 reported recommending for both adults and children. In 2023, 95 recommended for both adults and children, ten MST recommended vaccination for adults only, and one did not have a formal vaccination programme in place. In 2023, these medical conditions included but were not limited to: obesity, long-term aspirin use, HIV/AIDS, diabetes, as well as diseases of the heart, lungs, liver, and kidneys.

#### 3.2.2 Doses distributed

**Fig. 3:**
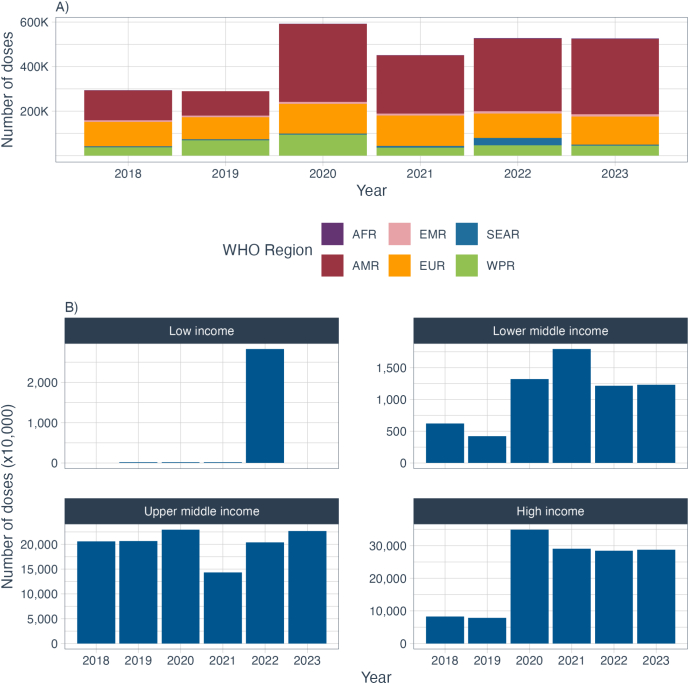
Summary of doses distributed (A) by WHO region and (B) by World Bank income group, between 2018 and 2023. The scale on the y axis of plot B varies by income group.

A large increase in the number of doses distributed globally can be seen in 2020 (up to 592 million doses). In 2022 and 2023, MST reported distributing over 525 million doses of seasonal influenza vaccines (Fig. 3). The number of MST that reported doses distributed and influenza coverage data (for at least one target group) increased from 80 in 2018 to 89 in 2023. An additional 17 MST reported the doses distributed, but did not report coverage data in 2023.

The total number of influenza vaccine doses distributed fluctuated over time and by income group, with a marked increase in 2020 in LMICs and HICs. Although the number of doses distributed returned to 2019 levels among LMIC, the number of doses distributed from 2021 onwards in HICs remained higher than in pre-COVID-19 periods. Reporting among LICs was heavily influenced by one MST that only reported in 2022. UMICs experienced a dip in distribution in 2021, before returning to pre-pandemic levels. In contrast, LMICs demonstrated a peak of distribution in 2021 (Fig. 3).

#### 3.2.3 Influenza vaccination coverage estimates

Globally, the number of MST with both policy recommendations and vaccination coverage data (numerator or pre-calculated coverage) for at least one of the WHO-recommended target groups remained steady with 91 MST in 2018 compared to 90 in 2023. Vaccination coverage was most often reported for older adults, which increased from 68 MST in 2018 to 81 in 2023. Similarly, vaccination coverage data reporting increased for HCWs from 54 MST to 59 MST in 2023. Slightly more MST reported coverage data for pregnant women over time (45 MST in 2018 compared to 48 in 2023) (Fig. 2B, Supplementary Table 4). A total of 12 MST reported vaccination coverage greater than 100% for at least one target group and at least one year; all were from one region (AMR).

**Fig. 4:**
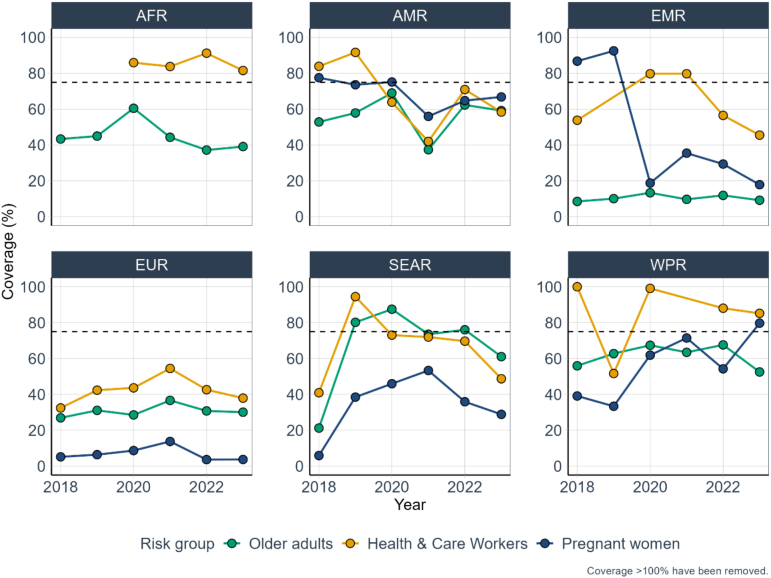
Median vaccination coverage, among MST who reported data for at least four years, by WHO region for three target groups: older adults, HCWs and pregnant women. Note: the horizontal dotted line represents the 75% vaccination coverage target for older adults and persons with chronic conditions set by WHO Member States in resolution 56.19. Number of MST included per WHO region (note that each MST may not have contributed data to all target groups): AFR: 2, AMR: 23, EMR: 4, EUR: 46, SEAR: 2 and WPR: 5.

Vaccination coverage per target group across MST that provided at least four years of data (*n* = 2 in AFR; *n* = 23 in AMR; *n* = 4 in EMR; *n* = 46 EUR; n = 2 in SEAR and *n* = 5 in WPR) showed large variations between regions. In four of the six WHO regions (AFR, EMR, EUR and WPR), the median vaccination coverage among reporting MST was highest among HCWs for four years, ranging from 82% to 91% in AFR; from 46% to 80% in EMR; from 32% to 54% in EUR and from 52% to 100% in WPR. In contrast, in SEAR, median vaccination coverage was highest among older adults from 2020 onwards. Vaccination coverage among pregnant women was consistently the lowest of the three target groups in EUR (peaking at 15% in 2021) and SEAR (peaking at 53% in 2021), but increased in WPR from 39% in 2018 to 80% in 2023 (Fig. 4).

##### 3.2.3.1 Global median coverage by target group (among MST reporting coverage data for at least four years)

The median coverage for older adults among the 74 MST with at least four years of data increased from 32% in 2018 to 39% in 2023. In comparison, the median coverage for HCWs among the 47 MST that provided data increased from 45% in 2018 to 48% in 2023. For pregnant women, the median coverage among the 44 MST reporting data increased from 38% in 2018 to 47% in 2023. Detailed information by WHO region is available in Fig. 4 and Supplementary Table 3.

##### 3.2.3.2 Comparison of reported data against other denominator sources

**Fig. 5:**
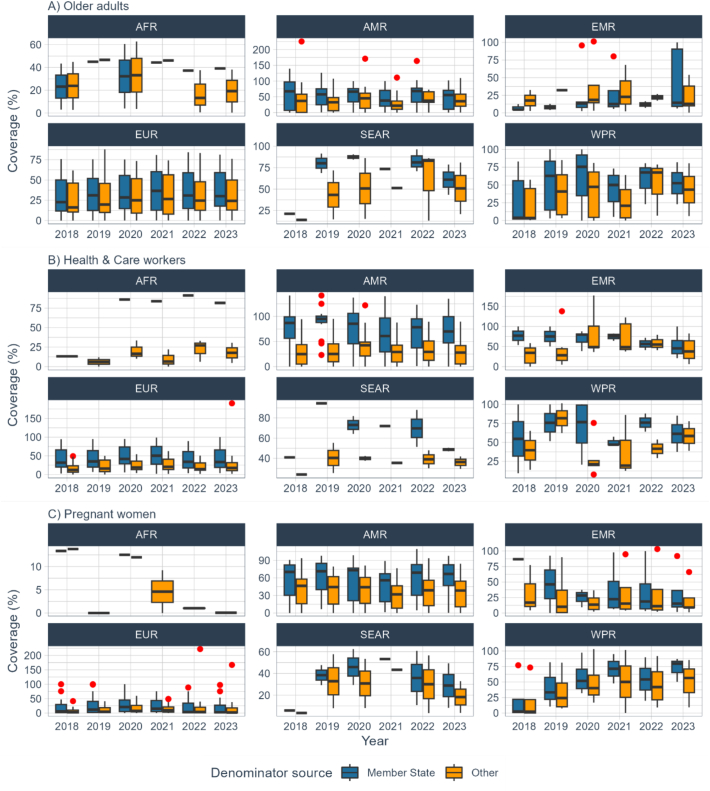
Comparison of median coverage per target group and reporting year, using data reported by MST (either pre-calculated coverage or numerator and denominators provided) and calculated using other data sources for denominators. Note: red points represent outliers; denominator sources are: Member State (provided by the MST); Other: UN population data for older adults, ILO for health workers and live births from UN population for pregnant women.

In all regions except AFR, the median coverage for older adults was lower when using the UN population data for the denominator than when using the reported coverage (either pre-calculated by each MST or calculated using numerators and denominators reported to JRF) (Supplementary Table 3, Fig. 5). We observed similar patterns when using the ILO data for HCWs and the UN data live births for pregnant women.

#### 3.2.4 Factors associated with higher coverage

In the linear mixed effects model looking at the entire time series, GDP was the only statically significant variable (*p* < 0.008), with a positive association (regression coefficient (β) = 1.26e−11, 95% confidence intervals: 9.25e−12 to 1.59e−11) and a modest fit (R^2^ = 0.154), meaning that MST with higher GDP tended to have a higher vaccination coverage among older adults.

When considering the linear regression model for 2023 only, GDP was again the only statistically significant variable (*p* = 0.006), with a positive association (regression coefficient (β) = 1.65e−11, 95% confidence intervals: 4.93e−12 to 2.82e−11), giving a similar interpretation to the results as for the previous model. The fit was also modest, with an adjusted R^2^ = 0.096). The other variables included in the model were not statistically significant.

## 4 Discussion

From 2018 to 2023, there have been increasing trends in the reporting of seasonal influenza vaccination and uptake of seasonal influenza vaccines, with regional variations. In four WHO regions, reported coverage was highest in HCWs, followed by older adults, and pregnant women. Only GDP was a statistically significant predictor of higher vaccination coverage in older adults globally.

Timely and complete influenza vaccination data are essential for policymakers to monitor performance, document progress, and make informed decisions about national influenza vaccination programmes (or other vaccination programmes with similar target groups). This information helps ministries of health, global health organizations, and partners learn from past campaigns to improve for the future. Annual monitoring and reporting are key to enabling access for vulnerable populations. These data are also used by WHO and partners to provide tailored technical and financial support to countries.

From 2018 to 2023, substantially more MST reported having influenza vaccination policies and programmes, and median influenza vaccination coverage rates for older adults, HCWs, and pregnant women increased. These increases may reflect several factors, including the provision of technical and financial support by WHO and partner organizations, [18], [19] global recognition of the value of seasonal influenza vaccination programmes for national pandemic preparedness, [20], [21] and the growing evidence base on the cost-effectiveness of seasonal influenza vaccination programmes for LMICs [22], [23], [24], [25].

However, this analysis highlights anomalies that occurred during the COVID-19 pandemic period. Globally, fewer MST responded to JRF during the COVID-19 pandemic, resulting in a lower percentage of MST reporting influenza vaccination coverage data. While several MST increased their influenza vaccine purchases to mitigate a potential surge in cases while their health systems were already strained from the COVID-19 pandemic [26], others reported decreased purchases for several reasons. In particular, 2019 data were requested from March to June 2020, a period in which most countries were actively engaging in the COVID-19 response, resulting in a lower response rate. MST can report and update historical data at any time.

Global and regional seasonal influenza vaccination coverage landscapes are complex. The aggregate summary figures feature wide ranges of minimum and maximum values across target groups and regions, demonstrating the need to consider regions separately. The regional coverage trends provide valuable information about the specific target groups where influenza vaccine uptake has improved or regressed, highlighting the value of regionally tailored approaches.

Several factors contribute to these regional differences, including both differences in national programmes (e.g., access to vaccines and service delivery capacity) and artificial differences due to data quality and reporting (e.g., incomplete data reported, out-of-date or mismatched target group size estimates). Factors such as (but not limited to) human resource constraints (particularly during the COVID-19 pandemic period), the transition from excel to electronic reporting of data, and changes in regional or national priorities could have impacted the reporting rate. In particular, national reporting of progress against programmatic coverage targets rather than immunization coverage and inaccurate denominators are persistent challenges. As few MST have a sufficient supply of seasonal influenza vaccines to cover the full target groups, they often establish programmatic coverage targets (the number of individuals they aim to immunize, considering the number of seasonal influenza vaccine doses the country has acquired). These targets provide a measurable benchmark against which to gauge progress. However, immunization coverage and progress against programmatic coverage targets are not the same measure. Immunization coverage is the total number of people vaccinated in the target group over the total number of people in the target group. Calculating progress against programmatic coverage targets typically uses a proportion of the overall target group size as the coverage calculation denominator rather than the overall target group population, which leads to reported coverage figures that are considerably greater than actual immunization coverage levels (often reaching over 100%). Relying on measures of progress against programmatic coverage goals can be misleading, as it masks real gaps in coverage, leaving target groups at risk. WHO's forthcoming manual on supporting seasonal influenza, COVID-19, and RSV immunization coverage calculation specifically addresses this issue to improve reported data quality.

Limitations of this analysis include data challenges and inconsistencies, resulting in incomplete or biased results. Many MST did not provide influenza vaccination coverage for all groups that they recommended for vaccination. Others provided incomplete data, for example, some MST reported only the doses administered (the numerator), but not the size of the target population (denominator), or provided coverage without a breakdown of the numerator or denominator. In addition, MST tend to have limited or no visibility on influenza vaccinations in the private sector and staff completing the JRF may not have complete data. As a result, the information included in this analysis primarily consists of vaccines distributed through the public sector. As noted in other articles, the lack of information on vaccination in the private sector, and incomplete reporting from MST on uptake, result in significant underreporting of global influenza vaccine doses used [6]. Similarly, at the national level, access to and use of only data on public sector vaccination results in an incomplete picture of the protection of priority groups and the total population. Lastly, the statistical analyses highlight the role of financing for seasonal influenza vaccination, with GDP the only variable statistically associated with increased coverage.

We recognize the potential impacts (both positive and negative) of the changes made to the JRF reporting questions between 2018 and 2023. The seasonal influenza vaccination JRF has been revised several times to simplify the reporting process for MST (e.g., streamlining the number of questions, providing instructions where relevant, etc.), but may have contributed to misunderstandings or confusion as questions changed. While the phrasing of the questions evolved, we anticipate that the shorter form is one of several factors contributing to more complete and timely reporting of the influenza vaccination data. To alleviate these roadblocks, WHO offers regular training on the JRF in multiple languages.

Other potential factors contributing to increased reporting include the transition of the JRF from an Excel to an online platform during this period. The online platform includes data validation rules, automatic calculation of coverage data when both the numerator and denominator are provided, and tooltips/instructions for how to complete questions. These features reduce human error and lighten the reporting burden for MST. A second potential factor leading to improved reporting and data quality is the systematic follow-up with MST. During the JRF submission and review processes, focal points at the WHO country, regional, and headquarters levels review the data, query potential data discrepancies, and request more information where needed. This validation process is critical to improve and maintain high data quality. For example, up to 25% of submitted data in one region was updated, as data issues were addressed, or previously missing information was provided. A third potential factor is the inclusion of influenza focal points in the JRF process. For MST where the influenza focal point sits outside the vaccination department, the influenza focal point can provide the relevant data on the JRF online platform, while still maintaining the vaccination department's ultimate responsibility for submission. WHO works closely with national counterparts to ensure familiarity with the JRF process and form.

In July 2024, WHO convened a meeting of internal stakeholders and partner organizations to discuss the global status of influenza vaccination and identify priorities for action. Recommendations from this meeting included the development of the global manual on monitoring and reporting vaccination coverage, identification of a potential process for estimating national coverage, and encouraging better use of existing tools for coverage analysis and programme optimization [27].

Seasonal influenza vaccination programmes have been leveraged during the 2009 A(H1N1)pdm09 and COVID-19 pandemic vaccine responses to enable the timely and efficient roll-out of pandemic vaccines. MST with existing influenza vaccination programmes rolled out the COVID-19 vaccines faster and reached higher vaccination coverage [28], [29], [30], [31]. Regional variation in influenza vaccine introduction and varying vaccination coverage trends from 2018 to 2023 highlight the different levels of pandemic vaccination capacity likely available in MST. Declines in vaccination coverage in some settings may reflect vulnerabilities in service continuity during health emergencies. These declines serve as indicators of potential gaps in the ability to sustain epidemic, pandemic, and essential immunization services during crises, informing future risk assessments and contingency planning.

Inversely, reported post-pandemic increases in coverage, particularly in certain high-priority groups, may signal recovery capacity and adaptive program strategies. Such trends can provide valuable insight into factors that facilitate rapid resumption or expansion of vaccination services, which is critical knowledge for building resilient delivery platforms that can be leveraged for future pandemics.

Efforts to increase vaccine uptake in at-risk groups should be prioritized in regions and MST reporting low coverage, especially in older adults and pregnant women. Low uptake of seasonal influenza vaccination among key risk groups, such as pregnant women in some regions, suggests a potential fragility in pandemic vaccine delivery to these populations. These gaps may pose challenges for the timely and equitable deployment of medical countermeasures during pandemics, highlighting the need for proactive investment in strengthening inclusive routine platforms and planning outreach strategies. In addition, fluctuations in health worker vaccination coverage may reflect operational or perceptual challenges that could impair a future pandemic response. As health workers are at elevated risk of exposure and central to service continuity in their routine practice as well as during influenza epidemics and pandemics, ensuring their protection through stable and accessible vaccination programmes must remain a core preparedness priority.

In the analysis of factors associated with achieving higher coverage rates for older adults, this manuscript documents a well-known equity issue for seasonal influenza vaccines. WHO, in collaboration with partners, works closely with interested countries to establish and strengthen seasonal influenza vaccination programmes. While the number of low- and middle-income countries introducing seasonal influenza vaccination has increased from 2018 to 2023, in part due to support from the WHO's Pandemic Influenza Preparedness Framework Partnership Contribution and The Task Force for Global Health’s Partnership for International Vaccines Initiative, the lack of financial support from Gavi, the vaccine alliance, impacts the ability of these countries to procure enough seasonal influenza vaccines or introduce vaccination. Gavi, the vaccine alliance, support for seasonal influenza vaccination of health workers and other at-risk groups would increase vaccine equity, reduce the burden of severe disease and deaths during annual epidemics, and strengthen pandemic preparedness.

Seasonal influenza vaccination programmes are used as a proxy for immunization system resilience and surge capacity during future respiratory pathogen pandemics [32]. When interpreted alongside contextual data, such as denominator quality, policy scope, and delivery modalities, these trends can inform preparedness indicators and help identify systems most capable of scaling during emergencies. Seasonal influenza vaccine availability and delivery infrastructure should not be interpreted in isolation. To meaningfully inform preparedness planning, coverage data should be assessed alongside indicators of policy implementation, regulatory processes, and waste management as factors that collectively shape a system's ability to sustain and scale vaccination during health emergencies.

## 5 Conclusion

Globally, more MST reported policies for and access to seasonal influenza vaccines between 2018 and 2023. Additionally, seasonal influenza vaccination coverage (both the number of MST reporting and the median coverage rates for target groups) improved. However, continued attention is needed to ensure countries can fully cover their priority groups. Increased coverage can protect vulnerable individuals from severe disease, reduce the number of influenza-associated deaths, and support national pandemic preparedness. Influenza vaccination coverage data helps MST and global health stakeholders monitor progress and understand how many lives are saved and hospitalizations are adverted through vaccination.

In addition, seasonal influenza vaccination monitoring and reporting enable countries to better prepare for and provide influenza vaccines to recommended groups during pandemic periods. To mitigate annual epidemics and prepare for future pandemics, WHO, partners, and countries need to communicate the value of seasonal influenza vaccination and secure sustainable funding for these important programmes.

## CRediT authorship contribution statement

**Margaux M.I. Meslé:** Writing – review & editing, Writing – original draft, Visualization, Methodology, Formal analysis, Data curation, Conceptualization. **Pernille Jorgensen:** Writing – review & editing, Writing – original draft, Visualization, Funding acquisition, Data curation, Conceptualization. **Amal Barakat:** Writing – review & editing, Data curation. **Belinda L. Herring:** Writing – review & editing, Data curation. **Francisco Nogareda:** Writing – review & editing, Data curation. **Marcela Contreras:** Writing – review & editing, Data curation. **Martha Velandia-Gonzalez:** Writing – review & editing, Data curation. **Pushpa Wijesinghe:** Writing – review & editing, Data curation. **Reena Doshi:** Writing – review & editing, Data curation. **Ioana Ghiga:** Writing – review & editing, Data curation. **Randie Gibson:** Writing – review & editing, Data curation. **Laure Dumolard:** Writing – review & editing, Data curation. **Donald J Brooks:** Writing – review & editing, Writing – original draft, Data curation. **Oluwatosin W. Akande:** Writing – review & editing. **Philipp Lambach:** Writing – review & editing. **Vanessa Cozza:** Writing – review & editing. **Ann C. Moen:** Writing – review & editing. **Stefano Tempia:** Writing – review & editing, Investigation. **Shoshanna Goldin:** Writing – review & editing, Writing – original draft, Visualization, Supervision, Project administration, Methodology, Data curation, Conceptualization.

## Funding

This work was supported by the Pandemic Influenza Preparedness Framework Partnership Contribution. The funders were not involved in the analysis or interpretation.

## Declaration of competing interest

The authors declare that they have no known competing financial interests or personal relationships that could have appeared to influence the work reported in this paper.

## Data Availability

Data will be made available on request.

## Footnotes

1 Note: Indonesia changed to WPR in 2025.

2 Influenza vaccines can be used from 6 months of age.
