# Supplementary material for: Influenza vaccine recommendations and coverage (2018–2023): a foundation for pandemic preparedness and response
Source: Vaccine. 2026 Apr 11;78:None. doi: 10.1016/j.vaccine.2026.128391 (PMC13058693; doi:10.1016/j.vaccine.2026.128391)
Supplement: Supplementary material [file mmc1.docx]

# Appendix to Influenza vaccine recommendations and coverage (2018-2023): a foundation for pandemic preparedness and response


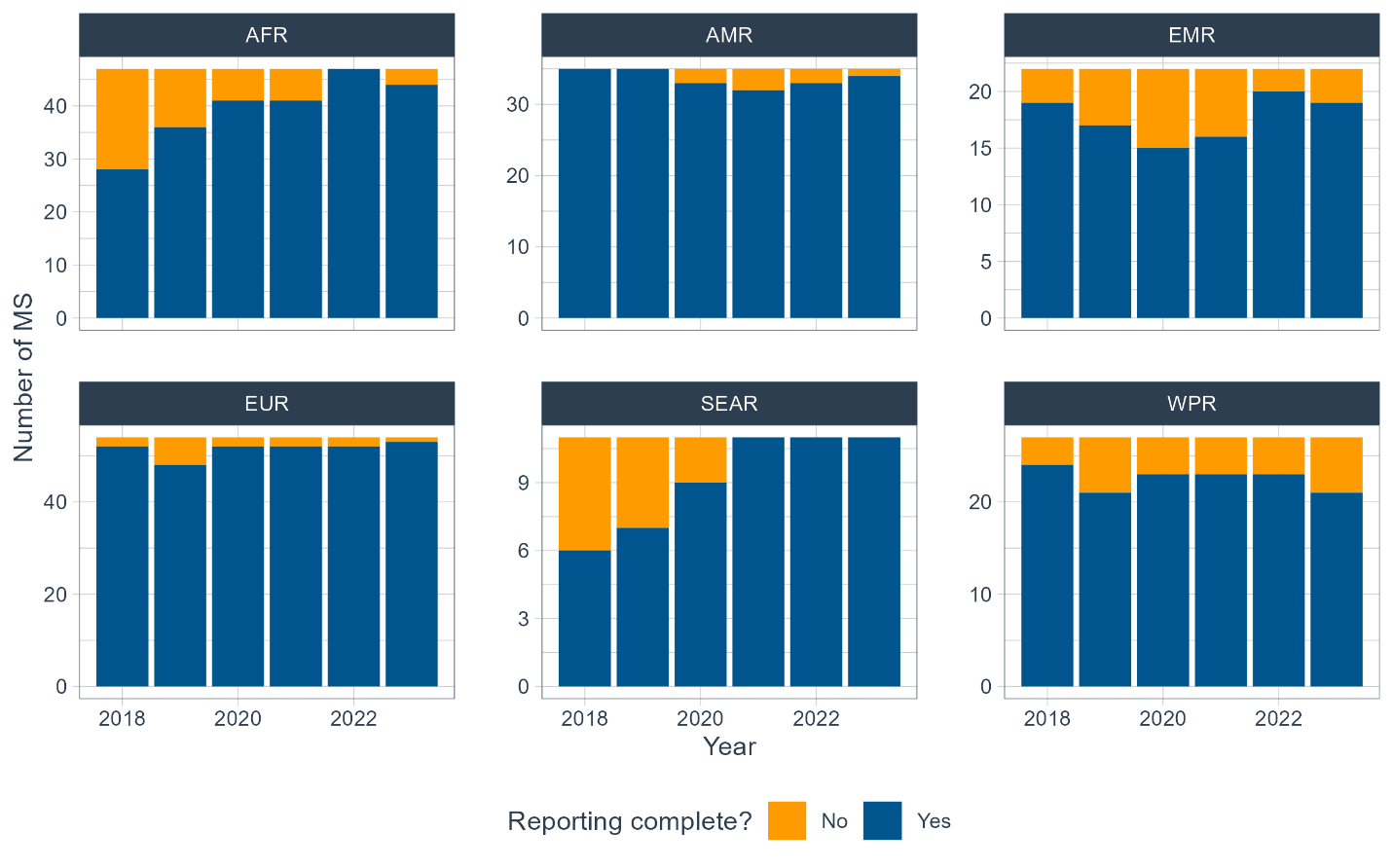


Supplementary Figure 1: Number of MST reporting to JRF in each WHO region by year. Note: the total number of MST in each region are: AFR: 47; AMR: 35; EMR: 22; EUR: 54; SEAR: 11; WPR: 27.

Supplementary table 1: Summary of Member States and territories included in each WHO Region.

| **WHO Region** | **Member States and territories included** |
| --- | --- |
| AFR  (n=47) | Algeria; Angola; Benin; Botswana; Burkina Faso; Burundi; Cabo Verde; Cameroon; Central African Republic; Chad; Comoros; Congo; Côte d’Ivoire; Democratic Republic of the Congo; Equatorial Guinea; Eritrea; Eswatini; Ethiopia; Gabon; Gambia; Ghana; Guinea; Guinea-Bissau; Kenya; Lesotho; Liberia ; Madagascar; Malawi; Mali; Mauritania; Mauritius; Mozambique; Namibia; Niger; Nigeria; Rwanda; Sao Tome and Principe; Senegal; Seychelles; Sierra Leone; South Africa; South Sudan; Togo; Uganda; United Republic of Tanzania; Zambia; Zimbabwe |
| AMR  (n=35) | Antigua and Barbuda; Argentina; Bahamas; Barbados; Belize; Bolivia (Plurinational State of); Brazil; Canada; Chile; Colombia; Costa Rica; Cuba; Dominica; Dominican Republic; Ecuador; El Salvador; Grenada; Guatemala; Guyana; Haiti; Honduras; Jamaica; Mexico; Nicaragua; Panama; Paraguay; Peru; Saint Kitts and Nevis; Saint Lucia; Saint Vincent and the Grenadines; Suriname; Trinidad and Tobago; United States of America; Uruguay; Venezuela (Bolivarian Republic of) |
| EMR  (n=22) | Afghanistan; Bahrain; Djibouti; Egypt; Iran (Islamic Republic of); Iraq; Jordan; Kuwait; Lebanon; Libya; Morocco; Oman; Pakistan; Qatar; Saudi Arabia; Somalia; Sudan; Syrian Arab Republic; Tunisia; United Arab Emirates; Yemen; the occupied Palestinian territory, including east Jerusalem |
| EUR  (n=54) | Albania; Andorra; Armenia; Austria; Azerbaijan; Belarus; Belgium; Bosnia and Herzegovina; Bulgaria; Croatia; Cyprus; Czechia; Denmark; Estonia; Finland; France; Georgia; Germany; Greece; Hungary; Iceland; Ireland; Israel; Italy; Kazakhstan; Kyrgyzstan; Latvia; Lithuania; Luxembourg; Malta; Monaco; Montenegro; Netherlands (Kingdom of the); North Macedonia; Norway; Poland; Portugal; Republic of Moldova; Romania; Russian Federation; San Marino; Serbia; Slovakia; Slovenia; Spain; Sweden; Switzerland; Tajikistan; Türkiye; Turkmenistan; Ukraine; United Kingdom of Great Britain and Northern Ireland; Uzbekistan; Kosovo (in accordance with UN Security Council resolution 1244 (1999)) |
| SEAR  (n=11) | Bangladesh; Bhutan; Democratic People's Republic of Korea; India; Indonesia; Maldives; Myanmar; Nepal; Sri Lanka; Thailand; Timor-Leste |
| WPR  (n=27) | Australia; Brunei Darussalam; Cambodia; China; Cook Islands; Fiji; Japan; Kiribati; Lao People's Democratic Republic; Malaysia; Marshall Islands; Micronesia (Federated States of); Mongolia; Nauru; New Zealand; Niue; Palau; Papua New Guinea; Philippines; Republic of Korea; Samoa; Singapore; Solomon Islands; Tonga; Tuvalu; Vanuatu; Viet Nam |

Supplementary table 2: Percentage and number (in brackets) with recommendations by target group, year, and WHO region of the MST that responded to the JRF.

| **Risk group** | **Region** | **Report year** | | | | | |
| --- | --- | --- | --- | --- | --- | --- | --- |
|  |  | **2018** | **2019** | **2020** | **2021** | **2022** | **2023** |
| Older adults | AFR (n=47) | 14% (4 / 28) | 6% (2 / 36) | 7% (3 / 41) | 10% (4 / 41) | 13% (6 / 47) | 11% (5 / 44) |
|  | AMR (n=35) | 77% (27 / 35) | 80% (28 / 35) | 85% (28 / 33) | 88% (28 / 32) | 88% (29 / 33) | 85% (29 / 34) |
|  | EMR (n=22) | 74% (14 / 19) | 65% (11 / 17) | 73% (11 / 15) | 75% (12 / 16) | 74% (14 / 19) | 79% (15 / 19) |
|  | EUR (n=54) | 94% (49 / 52) | 90% (43 / 48) | 94% (49 / 52) | 92% (48 / 52) | 90% (47 / 52) | 96% (51 / 53) |
|  | SEAR (n=11) | 17% (1 / 6) | 43% (3 / 7) | 22% (2 / 9) | 27% (3 / 11) | 27% (3 / 11) | 36% (4 / 11) |
|  | WPR (n=27) | 62% (15 / 24) | 57% (12 / 21) | 48% (11 / 23) | 48% (11 / 23) | 35% (8 / 23) | 43% (9 / 21) |
|  | Global (n=196) | 67% (110 / 164) | 60% (99 / 164) | 60% (104 / 173) | 61% (106 / 175) | 58% (107 / 185) | 62% (113 / 182) |
| Health & Care workers | AFR (n=47) | 7% (2 / 28) | 3% (1 / 36) | 10% (4 / 41) | 12% (5 / 41) | 13% (6 / 47) | 11% (5 / 44) |
|  | AMR (n=35) | 83% (29 / 35) | 89% (31 / 35) | 85% (28 / 33) | 91% (29 / 32) | 88% (29 / 33) | 88% (30 / 34) |
|  | EMR (n=22) | 79% (15 / 19) | 76% (13 / 17) | 67% (10 / 15) | 69% (11 / 16) | 68% (13 / 19) | 74% (14 / 19) |
|  | EUR (n=54) | 92% (48 / 52) | 90% (43 / 48) | 94% (49 / 52) | 96% (50 / 52) | 94% (49 / 52) | 100% (53 / 53) |
|  | SEAR (n=11) | 17% (1 / 6) | 43% (3 / 7) | 22% (2 / 9) | 36% (4 / 11) | 36% (4 / 11) | 27% (3 / 11) |
|  | WPR (n=27) | 50% (12 / 24) | 52% (11 / 21) | 35% (8 / 23) | 43% (10 / 23) | 35% (8 / 23) | 43% (9 / 21) |
|  | Global (n=196) | 65% (107 / 164) | 62% (102 / 164) | 58% (101 / 173) | 62% (109 / 175) | 59% (109 / 185) | 63% (114 / 182) |
| Pregnant women | AFR (n=47) | 7% (2 / 28) | -  (0 / 36) | 2% (1 / 41) | 7% (3 / 41) | 6% (3 / 47) | 9% (4 / 44) |
|  | AMR (n=35) | 80% (28 / 35) | 80% (28 / 35) | 79% (26 / 33) | 84% (27 / 32) | 85% (28 / 33) | 88% (30 / 34) |
|  | EMR (n=22) | 63% (12 / 19) | 41% (7 / 17) | 60% (9 / 15) | 62% (10 / 16) | 68% (13 / 19) | 74% (14 / 19) |
|  | EUR (n=54) | 88% (46 / 52) | 85% (41 / 48) | 94% (49 / 52) | 92% (48 / 52) | 90% (47 / 52) | 96% (51 / 53) |
|  | SEAR (n=11) | 17% (1 / 6) | 29% (2 / 7) | 22% (2 / 9) | 27% (3 / 11) | 27% (3 / 11) | 36% (4 / 11) |
|  | WPR (n=27) | 38% (9 / 24) | 43% (9 / 21) | 35% (8 / 23) | 39% (9 / 23) | 30% (7 / 23) | 43% (9 / 21) |
|  | Global (n=196) | 60% (98 / 164) | 53% (87 / 164) | 55% (95 / 173) | 57% (100 / 175) | 55% (101 / 185) | 62% (112 / 182) |
| Chronic population | AFR (n=47) | 11% (3 / 28) | 3% (1 / 36) | 7% (3 / 41) | 7% (3 / 41) | 13% (6 / 47) | 9% (4 / 44) |
|  | AMR (n=35) | 86% (30 / 35) | 86% (30 / 35) | 82% (27 / 33) | 84% (27 / 32) | 85% (28 / 33) | 74% (25 / 34) |
|  | EMR (n=22) | 68% (13 / 19) | 59% (10 / 17) | 73% (11 / 15) | 75% (12 / 16) | 74% (14 / 19) | 79% (15 / 19) |
|  | EUR (n=54) | 92% (48 / 52) | 90% (43 / 48) | 96% (50 / 52) | 96% (50 / 52) | 90% (47 / 52) | 96% (51 / 53) |
|  | SEAR (n=11) | 17% (1 / 6) | 43% (3 / 7) | 22% (2 / 9) | 27% (3 / 11) | 27% (3 / 11) | 36% (4 / 11) |
|  | WPR (n=27) | 50% (12 / 24) | 48% (10 / 21) | 35% (8 / 23) | 39% (9 / 23) | 30% (7 / 23) | 33% (7 / 21) |
|  | Global (n=196) | 65% (107 / 164) | 59% (97 / 164) | 58% (101 / 173) | 59% (104 / 175) | 57% (105 / 185) | 58% (106 / 182) |
| Children | AFR (n=47) | 11% (3 / 28) | 6% (2 / 36) | 7% (3 / 41) | 7% (3 / 41) | 4% (2 / 47) | 5% (2 / 44) |
|  | AMR (n=35) | 74% (26 / 35) | 74% (26 / 35) | 76% (25 / 33) | 72% (23 / 32) | 76% (25 / 33) | 74% (25 / 34) |
|  | EMR (n=22) | 63% (12 / 19) | 41% (7 / 17) | 53% (8 / 15) | 56% (9 / 16) | 53% (10 / 19) | 58% (11 / 19) |
|  | EUR (n=54) | 44% (23 / 52) | 42% (20 / 48) | 58% (30 / 52) | 52% (27 / 52) | 48% (25 / 52) | 62% (33 / 53) |
|  | SEAR (n=11) | 17% (1 / 6) | 29% (2 / 7) | 22% (2 / 9) | 27% (3 / 11) | 27% (3 / 11) | 27% (3 / 11) |
|  | WPR (n=27) | 46% (11 / 24) | 38% (8 / 21) | 43% (10 / 23) | 43% (10 / 23) | 35% (8 / 23) | 29% (6 / 21) |
|  | Global (n=196) | 46% (76 / 164) | 40% (65 / 164) | 45% (78 / 173) | 43% (75 / 175) | 39% (73 / 185) | 44% (80 / 182) |

Supplementary table 3: Summary of the percentage and number (in brackets) of MST who reported coverage data (numerator or coverage estimates) for the three target groups of interest and reporting year.

| **Target**  **group** | **Region** | **Report year** | | | | | |
| --- | --- | --- | --- | --- | --- | --- | --- |
|  |  | **2018** | **2019** | **2020** | **2021** | **2022** | **2023** |
| Older adults | AFR | 4% (2) | 4% (2) | 4% (2) | 4% (2) | 6% (3) | 4% (2) |
|  | AMR | 66% (23) | 71% (25) | 66% (23) | 60% (21) | 63% (22) | 66% (23) |
|  | EMR | 18% (4) | 18% (4) | 23% (5) | 18% (4) | 14% (3) | 27% (6) |
|  | EUR | 70% (38) | 74% (40) | 81% (44) | 81% (44) | 81% (44) | 91% (49) |
|  | SEAR | 9% (1) | 18% (2) | 18% (2) | 9% (1) | 27% (3) | 18% (2) |
|  | WPR | 22% (6) | 22% (6) | 22% (6) | 19% (5) | 11% (3) | 7% (2) |
|  | **Global** | **38% (74)** | **40% (79)** | **42% (82)** | **39% (77)** | **40% (78)** | **43% (84)** |
| Health & Care workers | AFR | 2% (1) | 4% (2) | 6% (3) | 6% (3) | 6% (3) | 4% (2) |
|  | AMR | 71% (25) | 60% (21) | 63% (22) | 57% (20) | 57% (20) | 69% (24) |
|  | EMR | 32% (7) | 32% (7) | 32% (7) | 23% (5) | 14% (3) | 32% (7) |
|  | EUR | 57% (31) | 52% (28) | 48% (26) | 54% (29) | 63% (34) | 65% (35) |
|  | SEAR | 9% (1) | 18% (2) | 18% (2) | 9% (1) | 18% (2) | 27% (3) |
|  | WPR | 11% (3) | 7% (2) | 19% (5) | 15% (4) | 11% (3) | 7% (2) |
|  | **Global** | **35% (68)** | **32% (62)** | **33% (65)** | **32% (62)** | **33% (65)** | **37% (73)** |
| Pregnant women | AFR | 2% (1) | 2% (1) | 2% (1) | 4% (2) | 4% (2) | 2% (1) |
|  | AMR | 71% (25) | 66% (23) | 60% (21) | 57% (20) | 60% (21) | 69% (24) |
|  | EMR | 14% (3) | 18% (4) | 18% (4) | 18% (4) | 18% (4) | 23% (5) |
|  | EUR | 44% (24) | 43% (23) | 46% (25) | 43% (23) | 48% (26) | 52% (28) |
|  | SEAR | 9% (1) | 18% (2) | 18% (2) | 9% (1) | 18% (2) | 18% (2) |
|  | WPR | 15% (4) | 15% (4) | 15% (4) | 11% (3) | 15% (4) | 11% (3) |
|  | **Global** | **30% (58)** | **29% (57)** | **29% (57)** | **27% (53)** | **30% (59)** | **32% (63)** |

Supplementary table 4: Summary comparison of median coverage for three target groups, per WHO region and JRF reporting year, using coverage data reported by MST in comparison to other denominator sources.

| **Risk group** | **Region** | **Denominator source** | **Median coverage (%) by report year** | | | | | |
| --- | --- | --- | --- | --- | --- | --- | --- | --- |
|  |  |  | **2018** | **2019** | **2020** | **2021** | **2022** | **2023** |
| Older adults | AFR | MST | 23 | 45 | 32 | 44 | 37 | 39 |
|  |  | Other sources | 24 | 47 | 33 | 46 | 13 | 19 |
|  | AMR | MST | 68 | 58 | 67 | 38 | 69 | 56 |
|  |  | Other sources | 37 | 33 | 45 | 22 | 38 | 36 |
|  | EMR | MST | 4.6 | 7.4 | 13 | 13 | 12 | 14 |
|  |  | Other sources | 18 | 32 | 18 | 23 | 22 | 13 |
|  | EUR | MST | 23 | 31 | 28 | 37 | 31 | 30 |
|  |  | Other sources | 16 | 18 | 25 | 27 | 26 | 24 |
|  | SEAR | MST | 21 | 80 | 87 | 74 | 81 | 61 |
|  |  | Other sources | 14 | 43 | 51 | 51 | 83 | 51 |
|  | WPR | MST | 3.9 | 63 | 75 | 50 | 68 | 52 |
|  |  | Other sources | 3.6 | 41 | 47 | 21 | 68 | 43 |
| Health & Care Workers | AFR | MST | - | - | 86 | 84 | 91 | 82 |
|  |  | Other sources | 13 | 6.0 | 17 | 6.5 | 27 | 18 |
|  | AMR | MST | 87 | 95 | 85 | 61 | 78 | 70 |
|  |  | Other sources | 25 | 25 | 43 | 29 | 29 | 28 |
|  | EMR | MST | 77 | 75 | 80 | 76 | 57 | 46 |
|  |  | Other sources | 34 | 28 | 49 | 48 | 55 | 38 |
|  | EUR | MST | 32 | 42 | 42 | 50 | 34 | 33 |
|  |  | Other sources | 12 | 16 | 19 | 21 | 16 | 18 |
|  | SEAR | MST | 41 | 94 | 73 | 72 | 70 | 49 |
|  |  | Other sources | 24 | 40 | 40 | 35 | 39 | 36 |
|  | WPR | MST | 55 | 76 | 77 | 47 | 76 | 61 |
|  |  | Other sources | 40 | 82 | 21 | 19 | 42 | 58 |
| Pregnant women | AFR | MST | 13 | - | 13 | - | - | - |
|  |  | Other sources | 14 | 0.0 | 12 | 4.6 | 1.0 | 0.1 |
|  | AMR | MST | 70 | 71 | 73 | 56 | 69 | 67 |
|  |  | Other sources | 46 | 44 | 44 | 32 | 39 | 38 |
|  | EMR | MST | 87 | 46 | 28 | 22 | 19 | 15 |
|  |  | Other sources | 17 | 10 | 14 | 15 | 11 | 9.0 |
|  | EUR | MST | 6.0 | 21 | 21 | 15 | 3.9 | 2.4 |
|  |  | Other sources | 2.9 | 4.5 | 7.4 | 9.7 | 3.8 | 1.8 |
|  | SEAR | MST | 5.8 | 38 | 46 | 53 | 36 | 29 |
|  |  | Other sources | 3.6 | 33 | 31 | 43 | 30 | 18 |
|  | WPR | MST | 2.5 | 33 | 52 | 71 | 54 | 80 |
|  |  | Other sources | 2.3 | 24 | 40 | 50 | 42 | 57 |
| Sources used for denominators are UN population for older adults (matching age groups reported by MS), ILO for HW and UN population live births for pregnant women. | | | | | | | | |
